# Strengthening capacity of health workers to diagnose birth defects in Ugandan hospitals from 2015 to 2021

**DOI:** 10.1186/s12909-023-04760-w

**Published:** 2023-10-13

**Authors:** Joyce Namale-Matovu, Ronald Kusolo, Robert Serunjogi, Linda Barlow-Mosha, Daniel Mumpe-Mwanja, Natalia Niombi, Dennis Kalibbala, Dhelia Williamson, Diana Valencia, Cynthia A. Moore, Kenneth Mwambi, Lisa J. Nelson, Phoebe Monalisa Namukanja-Mayambala, Jennifer L. Williams, Cara T. Mai, Yan Ping Qi, Philippa Musoke

**Affiliations:** 1 Makerere University, Johns Hopkins University Research Collaboration (MU-JHU), P.O. Box 23491, Kampala, Uganda; 2 Kawempe National Referral Hospital, Kampala, Uganda; 3 Division of Global HIV and TB, Centers for Disease Control and Prevention (CDC), Atlanta, GA USA; 4 National Center on Birth Defects and Developmental Disabilities, CDC, Atlanta, GA USA; 5 Goldbelt Professional Services, LLC, Chesapeake, VG USA; 6 Division of Global HIV and TB, US CDC, Kampala, Uganda; 7 Department of Pediatrics, Makerere University College of Health Sciences, Kampala, Uganda

**Keywords:** Birth defects, Surveillance, Midwives, Uganda, Capacity building

## Abstract

**Background:**

Limited diagnostic capabilities, resources and health worker skills have deterred the advancement of birth defects surveillance systems in most low- and middle-income countries (LMICs). Empowering health workers to identify and diagnose major external birth defects (BDs) is crucial to establishing effective hospital-based BD surveillance. Makerere University-Johns Hopkins University (MU-JHU) Research Collaboration BD Surveillance System consists of three diagnostic levels: (1) surveillance midwives, (2) MU-JHU clinical team, and (3) U.S. Centers for Disease Control and Prevention (CDC) birth defects subject matter experts (SMEs) who provide confirmatory diagnosis. The diagnostic concordance of major external BDs by surveillance midwives or MU-JHU clinical team with CDC birth defects SMEs were estimated.

**Methods:**

Study staff went through a series of trainings, including birth defects identification and confirmation, before surveillance activities were implemented. To assess the diagnostic concordance, we analyzed surveillance data from 2015 to 2021 for major external BDs: anencephaly, iniencephaly, encephalocele, spina bifida, craniorachischisis, microcephaly, anophthalmia/microphthalmia, anotia/microtia, cleft palate alone, cleft lip alone, cleft lip with cleft palate, imperforate anus, hypospadias, talipes equinovarus, limb reduction, gastroschisis, and omphalocele. Positive predictive value (PPV) as the proportion of BDs diagnosed by surveillance midwives or MU-JHU clinical team that were confirmed by CDC birth defects SMEs was computed. PPVs between 2015 and 2018 and 2019–2021 were compared to assess the accuracy of case diagnosis over time.

**Results:**

Of the 204,332 infants examined during 2015–2021, 870 infants had a BD. Among the 1,245 BDs identified, 1,232 (99.0%) were confirmed by CDC birth defects SMEs. For surveillance midwives, PPV for 7 of 17 BDs was > 80%. For the MU-JHU clinical team, PPV for 13 of 17 BDs was > 80%. Among surveillance midwives, PPV improved significantly from 2015 to 2018 to 2019–2021, for microcephaly (+ 50.0%), cleft lip with cleft palate (+ 17.0%), imperforate anus (+ 30.0%), and talipes equinovarus (+ 10.8%). Improvements in PPV were also observed among MU-JHU clinical team; however, none were significant.

**Conclusion:**

The diagnostic accuracy of the midwives and clinical team increased, highlighting that BD surveillance, by front-line health care workers (midwives) in LMICs is possible when midwives receive comprehensive training, technical support, funding and continuous professional development.

**Supplementary Information:**

The online version contains supplementary material available at 10.1186/s12909-023-04760-w.

## Background

A hospital-based birth defects (BD) surveillance system captures birth defects that occur in selected hospitals within a defined geographic area [1]. Most low- and middle-income countries do not have accurate data on the prevalence of birth defects due to a lack of diagnostic capabilities, resources, and awareness of available services, as well as the absence of a birth defects surveillance system [2, 3]. Lack of routine, systematically-collected, accurate data can inhibit the ability to develop, monitor, and assess birth defects prevention and intervention activities [4].

Integrating a successful birth defects surveillance system into an existing hospital structure can be a daunting task. Challenges include, integration of surveillance activities in busy delivery units, burden on staff to conduct additional activities, such as; reporting BD data, recording the clinical presentation of any identified birth defect [5], rigorous reporting structures/hierarchy for surveillance midwives conducting birth defect activities, and limited space, equipment and supplies to successfully conduct surveillance activities [6]. Strengthening health workers’ capacity for identification and diagnosis of birth defects is crucial. It fosters development of competencies and skills needed to correctly diagnose birth defects and establish an effective hospital-based birth defects surveillance program.

The objectives of this study were to describe the process for strengthening capacity of health workers to diagnose external major birth defects in four large Ugandan hospitals and to examine the concordance of these diagnoses to those of birth defects subject matter experts (SMEs) at the U.S. Centers for Disease Control and Prevention (CDC) to assess the competence of surveillance staff in diagnosing birth defects and whether or not this improved over time. Positive predictive value (PPV) between 2015 and 2018 and 2019–2021 for both surveillance midwives and the Makerere University-Johns Hopkins University (MU-JHU) Research Collaboration clinical team were compared.

## Methods

In 2015, Makerere University–Johns Hopkins University Research Collaboration, in collaboration with CDC, implemented a hospital-based birth defects surveillance system in Kampala, Uganda. This was the first birth defects surveillance system in Uganda. The surveillance system was designed to provide accurate prevalence estimates of 17 major external birth defects (i.e., anencephaly, iniencephaly, encephalocele, spina bifida, craniorachischisis, microcephaly, anophthalmia/microphthalmia, anotia/microtia, cleft palate alone, cleft lip alone, cleft lip with cleft palate, imperforate anus, hypospadias, talipes equinovarus, limb reduction, gastroschisis, and omphalocele) [7] at four large hospitals: Kawempe National Referral Hospital; Mengo Hospital; St. Francis Hospital, Nsambya; and Uganda Martyrs Hospital, Lubaga. Definitions of these birth defects are presented in Table 1 of the additional file. All birth defects were included - no exclusions were made based on diagnosis of a syndrome. Results are presented by birth defect and not by infant i.e. if an infant had more than one defect, then each defect was included as a separate case for that particular defect. The MU-JHU clinical team consisted of two pediatricians (the principal investigator and a co-investigator), one physician serving as the program manager and one hospital liaison coordinator.

### Process for strengthening capacity of health workers

The initial training for MU-JHU clinical team included an online 7-week pre-course training and a 5-day in-person birth defects surveillance workshop conducted in 2015 by the International Clearinghouse for Birth Defects Surveillance and Research (ICBDSR) and the U.S. CDC [8]. The workshop focused on increasing awareness of the importance of birth defects surveillance, the need for establishing a comprehensive program to collect accurate and timely data, and the utilization of these data to plan for implementation and evaluation of birth defects prevention programs [8].

The trained MU-JHU clinical team and data managers collaborated with CDC to further develop the surveillance protocol, standard operating procedures (SOPs), data collection logistics, and data collection forms. Additional surveillance study staff (i.e., surveillance midwives, research assistants, study consultants) were recruited to conduct the initial examination of all newborns and collect data, support quality control/assurance, and conduct bedside confirmation of birth defects at the hospitals.

Surveillance study staff were trained by CDC and the MU-JHU clinical team on the study protocol, SOPs, good clinical practice, data collection, as well as the systematic process of examining all informative births for major external birth defects [6]. Additional engagement to further strengthen capacity of health workers to diagnose birth defects included: clinical workshop trainings [8], mentoring/coaching, customized birth defect protocol trainings, hands-on trainings with support from the MU-JHU clinical team/supervisors/experienced surveillance staff, data managers, and feedback from the MU-JHU clinical team to surveillance midwives on birth defects cases. Furthermore, formalized in-house continuous medical trainings and hands-on trainings, led by MU-JHU clinical team, were organized to upgrade professional skills and competence in diagnosing birth defects, among surveillance midwives.

Diagnosis and verification of the birth defects are done at several levels to ensure accuracy. First, the surveillance midwives conduct the initial examination of each newborn to identify any external birth defects. Newborns are examined when they are clinically stable and external birth defects are documented in the relevant study logs at the hospitals. Data are collected electronically on android-based tablets using the Open Data Kit (ODK) platform [5]. Next, selected study staff (i.e., study investigators, program manager/clinical consultants, hospital liaison coordinator, nurse coordinator and data managers) are notified about the identified birth defect through a phone call or text message. The program manager/clinical consultant, who can be an on-call clinician, pediatrician or obstetrician-gynecologist, performs bedside examination of the newborn to confirm the presence of any birth defects and determine if the birth defect is of interest to the study. If the program manager/clinical consultant on-call confirms that the birth defect is of interest, maternal informed consent is obtained by the surveillance midwife to obtain photographs of the fetus’ or newborn’s birth defect, which is then used for case review and classification by the MU-JHU clinical team and CDC birth defects SME.

In all hospitals, if the program manager/clinical consultant is not available to perform bedside confirmation or if consent for photographs of the case is not obtained, the surveillance midwife draws an illustration of the defect and writes a brief narrative description of the condition. This visual data along with the surveillance information are then entered into a tablet for further review by another surveillance midwife and then submitted to the MU-JHU central server. Thereafter, the birth defects records are extracted by the data managers and shared with the MU-JHU clinical team for review and classification.

This process is followed in all hospitals except at Kawempe National Referral Hospital, a high volume government hospital where up to 70 mothers give births daily and close to 24,000 babies are delivered annually [9]. The surveillance midwives at this referral hospital were further trained to conduct bedside confirmation of birth defects in absence of a program manager/on-call clinical consultant. If a surveillance midwife identifies a birth defect, she informs a second midwife on duty to also examine the baby. The birth defect is confirmed if there is agreement by the two midwives. If there is disagreement, a third midwife examines the newborn and a diagnosis is established based on two concurring assessments. If all three midwives disagree on the diagnosis, then the examination findings and all data (photographs, illustrations and narrative description) are submitted to the MU-JHU central server, and thereafter the birth defects records are extracted by the data managers and shared with the MU-JHU clinical team for review and classification [5].

After cases are reviewed by the MU-JHU clinical team, the compilation of birth defects pictures/illustrations, descriptions, diagnoses, and coding are sent to CDC birth defects SMEs by data mangers through encrypted files. Feedback from CDC birth defects SMEs along with any corrections to the diagnoses or coding are sent back to the MU-JHU clinical team as well as the surveillance midwives at the hospital level. Birth defects diagnoses are coded using the International Classification of Diseases (ICD) 10 classification system by the Royal College of Paediatrics and Child Health (RCPCH). All care of live newborns with birth defects is provided through routine care by the hospitals, which includes referral to specialists when available [3].

### Statistical analysis

Birth defects surveillance data were used to calculate PPV of birth defects diagnosis at each level of review (i.e., surveillance midwives and MU-JHU clinical team), using the CDC subject matter experts’ review as the “gold standard.” PPV was calculated to assess how well the surveillance midwives and MU-JHU clinical team diagnosed and classified the identified birth defects compared to the CDC birth defects SME review. The PPVs for surveillance midwives were calculated as the proportion of a given birth defect diagnosed by the surveillance midwives that was confirmed by the CDC birth defects SME review. The PPVs for the MU-JHU clinical team were calculated as the proportion of a given birth defect classified by the MU-JHU clinical team that was confirmed by the CDC birth defects SME review. For both surveillance midwives and MU-JHU clinical team, the PPVs in the earlier periods of the surveillance activities (2015–2018) were compared to PPVs of the later periods (2019–2021) using the two-sided proportion test where the numbers were quite high. Where the occurrences of defects were rare, like craniorachischisis, a Fisher’s exact test was used to determine if classification improved over time, given the ongoing capacity building and additional staff trainings. STATA version 17 software (StataCorp. 2017. College Station, TX: StataCorp LLC) was used for analysis.

## Results

Birth defects ascertainment was performed by 202 surveillance midwives at the hospital level and reviewed by four MU-JHU clinical team members (Table 1) and two birth defects SMEs at CDC. All four MU-JHU clinical team members had ≥ 4 years of surveillance experience. Among surveillance midwives, over 90% (184/202) had ≥ 2 years and 62% (125/202) had ≥ 4 years of surveillance experience (Table 1). Surveillance midwives examined 204,332 newborns during August 2015–December 2021. A total of 1,245 birth defects were identified, and 99% of these (1,232) were reviewed and confirmed by CDC birth defects SMEs. The remaining 1% were reviewed but unconfirmed due to poor photograph quality or insufficient supporting information.

**Table 1:** Surveillance staff from 2015 to end of 2021, Kampala, Uganda

Cadre	Trained on surveillance	Staff with 1 year’s surveillance experience	Staff with 2 years surveillance experience	Staff with 3 years surveillance experience	Staff with 4 years and more surveillance experience
**MU-JHU Clinical Team**					
Principal/co-investigator	2				2
Program Manager	1				1
Hospital Liaison Coordinator	1				1
**Other Surveillance Study Staff**					
Nurse coordinator	3	1	1	1	
Surveillance Midwives	202	18	29	30	125
Clinical consultants	13	4	2	0	7
Data Managers	4	1	1	1	1

From 2015 to 2023, the surveillance midwives had a PPV of > 80% for 7 external birth defects: hypospadias (95%), omphalocele (90%), spina bifida (89%), anencephaly (87%), imperforate anus (86%), cleft lip with palate (82%) and gastroschisis (81%) (Table 2). Birth defects with the lowest PPVs, for surveillance midwives, were craniorachischisis (60%), encephalocele (66%) and cleft lip only (67%). The MU-JHU clinical team had a PPV of > 85% for 13 birth defects: anophthalmia/microphthalmia (100%), anotia/microtia (100%), iniencephaly (100%), hypospadias (98%), cleft lip only (97%), imperforate anus (93%), cleft lip with cleft palate (92%), talipes equinovarus (92%), spina bifida (91%), omphalocele (89%), anencephaly (89%), gastroschisis (88%), and limb reduction deficiencies (87%); while the lowest PPVs were observed for craniorachischisis (50%) and microcephaly (68.%) (Table 3).

**Table 2:** Positive predictive value (PPV) for diagnoses by surveillance midwives compared to birth defects subject matter experts at Centers for Disease Control and Prevention, August 2015 to December 2021, Kampala, Uganda

Birth Defects	Number identified by surveillance midwives	Number confirmed by CDC subject matter experts	PPV (%)
Malformations of the central nervous system			
Anencephaly	54	47	87.0
Craniorachischisis	5	3	60.0
Iniencephaly	0	0	NA
Encephalocele	32	21	65.6
Spina bifida	103	92	89.3
Microcephaly	21	15	71.4
Malformations of eye and ear			
Anophthalmia/ Microphthalmia	29	21	72.4
Anotia/Microtia	35	27	77.1
Orofacial clefts			
Cleft palate only	20	15	75.0
Cleft lip only	33	22	66.7
Cleft lip with cleft palate	72	59	81.9
Malformations of the digestive system			
Imperforate anus	21	18	85.7
Malformations of the genital organ			
Hypospadias (Male)	192	183	95.3
Malformations of the limbs			
Talipes Equinovarus/clubfoot	269	202	75.1
Limb reduction deficiencies	75	55	73.3
Abdominal wall defects			
Gastroschisis	42	34	81.0
Omphalocele	61	55	90.2

**Table 3:** Positive predictive value (PPV) for MU-JHU clinical team diagnoses compared to birth defects subject matter experts at Centers for Disease Control and Prevention, August 2015 to December 2021, Kampala, Uganda

Birth Defects	Number classified by MU-JHU clinical team	Number confirmed by CDC subject matter experts	PPV (%)
Malformations of the central nervous system			
Anencephaly	53	47	88.7
Craniorachischisis	4	2	50.0
Iniencephaly	1	1	100.0
Encephalocele	30	23	76.7
Spina bifida	115	105	91.3
Microcephaly	25	17	68.0
Malformations of eye and ear			
Anophthalmia/ Microphthalmia	28	28	100.0
Anotia/Microtia	53	53	100.0
Orofacial clefts			
Cleft palate only	32	23	71.9
Cleft lip only	33	32	97.0
Cleft lip with cleft palate	91	84	92.3
Malformations of the digestive system			
Imperforate anus	30	28	93.3
Malformations of the genital organ			
Hypospadias (Male)	209	205	98.1
Malformations of the limbs			
Talipes Equinovarus/clubfoot	272	250	91.9
Limb reduction deficiencies	108	94	87.0
Abdominal wall defects			
Gastroschisis	42	37	88.1
Omphalocele	85	76	89.4

Generally, higher PPVs were observed among surveillance midwives during the later period (2019–2021) compared to the earlier period of when the surveillance system began (2015–2018) (Table 4). Among surveillance midwives, PPV increased significantly for microcephaly (50–100%, p = 0.019), cleft lip with cleft palate (72.7–89.7%, p = 0.031), imperforate anus (70–100%, p = 0.025), and talipes equinovarus (70.9–81.7%, p = 0.023). A non-significant increase in PPV among surveillance midwives was observed for 10 other birth defects, including: anencephaly (85.7–89.5%, p = 0.347), encephalocele (61.1–71.4%,p = 0.271), spina bifida (85.7–95%, 0.069), anophthalmia/microphthalmia (71.4–73.3%, p = 0.454), anotia/microtia (76.2–78.6%, p = 0.435), cleft palate only (70.6–100%, p = 0.540), hypospadias (93.9–97.4%, p = 0.125), limb reduction deficiencies (68.2–80.6%, p = 0.115), gastroschisis (73.9–89.5%, p = 0.101) and omphalocele (86.7–100%, p = 0.062). For the MU-JHU clinical team, there was an increase in PPV from 2019 to 2021 compared to 2015–2018 for 5 birth defects, including: anencephaly (88.2–89.5%, p = 0.446), encephalocele (72.2–83.3%, p = 0.240), craniorachischisis (33.3–100%, p = 0.999), cleft lip with cleft palate (88.4–95.8%, p = 0.091), imperforate anus (92.3–94.1%, p = 0.422); although none were statistically significant. There were also decreases in PPV for nine birth defects, none of which were statistically significant (Table 5).

**Table 4:** Comparison of PPV of birth defects diagnoses by surveillance midwives between 2015–2018 and 2019–2021, Kampala, Uganda

Birth Defects	2015–2018		2019–2021			p-value
	Frequency*	PPV (%)	Frequency^*^	PPV (%)	PPV Difference	
Malformations of the central nervous system						
Anencephaly	30/35	85.7	17/19	89.5	+ 3.8	0.347
Craniorachischisis	2/3	66.7	1/2	50.0	-16.7	0.999
Iniencephaly	0/0	NA	0/0	NA	NA	NA
Encephalocele	11/18	61.1	10/14	71.4	+ 10.3	0.271
Spina bifida	54/63	85.7	38/40	95.0	+ 9.3	0.069
Microcephaly	6/12	50.0	9/9	100.0	+ 50.0	0.019
Malformations of eye and ear						
Anophthalmia/ Microphthalmia	10/14	71.4	11/15	73.3	+ 1.9	0.454
Anotia/Microtia	16/21	76.2	11/14	78.6	+ 2.4	0.435
Orofacial clefts						
Cleft palate only	12/17	70.6	3/3	100.0	+ 29.4	0.540
Cleft lip only	13/18	72.2	9/15	60.0	-12.0	0.771
Cleft lip with cleft palate	24/33	72.7	35/39	89.7	+ 17.0	0.031
Malformations of the digestive system						
Imperforate anus	7/10	70.0	11/11	100.0	+ 30.0	0.025
Malformations of the genital organ						
Hypospadias (Male)	107/114	93.9	76/78	97.4	+ 3.5	0.125
Malformations of the limbs						
Talipes Equinovarus/clubfoot	117/165	70.9	85/104	81.7	+ 10.8	0.023
Limb reduction deficiencies	30/44	68.2	25/31	80.6	+ 12.4	0.115
Abdominal wall defects						
Gastroschisis	17/23	73.9	17/19	89.5	+ 15.6	0.101
Omphalocele	39/45	86.7	16/16	100.0	+ 13.3	0.062

*Number confirmed by CDC subject matter experts to be correctly diagnosed/ Number diagnosed by surveillance midwives for a given birth defect.

**Table 5:** Comparison of PPV of birth defects diagnoses by MU-JHU clinical team between 2015–2018 and 2019–2021, Kampala, Uganda

Birth Defects	2015–2018		2019–2021			p-value
	Frequency*	PPV (%)	Frequency*	PPV (%)	PPV Difference	
Malformations of the central nervous system						
Anencephaly	30/34	88.2	17/19	89.5	+ 1.3	0.446
Craniorachischisis	1/3	33.3	1/1	100.0	+ 66.7	0.999
Iniencephaly	0/0	NA	1/1	100.0	NA	NA
Encephalocele	13/18	72.2	10/12	83.3	+ 11.1	0.240
Spina bifida	61/65	93.8	44/50	88.0	-5.8	0.865
Microcephaly	10/14	71.4	7/11	63.6	-7.8	0.661
Malformations of eye and ear						
Anophthalmia/ Microphthalmia	17/17	100.0	11/11	100.0	0.0	NA
Anotia/Microtia	32/32	100.0	21/21	100.0	0.0	NA
Orofacial clefts						
Cleft palate only	15/20	75.0	8/12	66.7	-8.3	0.694
Cleft lip only	16/16	100.0	16/17	94.1	-5.9	0.838
Cleft lip with cleft palate	38/43	88.4	46/48	95.8	+ 7.4	0.091
Malformations of the digestive system						
Imperforate anus	12/13	92.3	16/17	94.1	+ 1.8	0.422
Malformations of the genital organ						
Hypospadias (Male)	130/131	99.2	75/78	96.2	-3.0	0.942
Malformations of the limbs						
Talipes Equinovarus/clubfoot	143/152	94.1	107/120	89.2	-4.9	0.930
Limb reduction deficiencies	48/54	88.9	46/54	85.2	-3.7	0.717
Abdominal wall defects						
Gastroschisis	20/22	90.9	17/20	85.0	-5.9	0.723
Omphalocele	54/58	93.1	22/27	81.5	-11.6	0.948

*Number confirmed by CDC subject matter experts to be correctly diagnosed/ Number diagnosed by MU-JHU central review team for a given birth defect.

## Discussion

Successful birth defects surveillance programs require skilled and competent staff to identify and diagnose birth defects rapidly and accurately. The importance of birth defect capacity building has been reported in other resource limited settings [9]. For birth defects surveillance systems to provide quality data, comprehensive training, capacity building, and mentorship of health care workers and implementing staff are of the utmost importance [10]. To further strengthen surveillance capacity for accurate birth defects diagnoses, a mobile application for data collection could be used to assist with birth defect identification, description and coding. This tool would be invaluable as staff can access it in real-time as they are examining the baby.

In Uganda, strengthening and building capacity among different cadres involved with surveillance activities has been done through organized workshops with international birth defects experts, formalized in-house continuous medical trainings and hands on trainings, and self-learning. In addition, continuous quality improvement activities were implemented to address data quality gaps and ensure the accuracy and completeness of every surveillance record. Several quality control measures were implemented including real-time quality control by surveillance midwives before data submissions, review of submitted data for accuracy and completeness by data managers before inclusion in the study database, and supportive supervision by trained senior surveillance study staff and the MU-JHU clinical team. Accuracy of birth defects diagnosis was positively associated with increasing use of readily available birth defect documentation tools, particularly; the ICD-10 form for standardized coding of birth defects, and the quality control verification form for checking completeness of data variables [11].

One limitation faced by this surveillance program was the high turnover (37%) among surveillance midwives, as it is not unusual for midwives to change hospitals in resource-limited settings like Uganda [12]. However, the training and skills these midwives gained from working in birth defects surveillance will be taken to other hospitals, which can only strengthen the identification and classification of birth defects in Uganda.

Overall, results from this study showed that the MU-JHU clinical team had a higher PPV for more birth defects than the surveillance midwives. This may have been because there was no turnover in the clinical team at MU-JHU and that the team worked collaboratively, to review the information provided on identified birth defects. A second limitation was the low prevalence of certain birth defects in the surveillance system. The lowest PPV for the MU-JHU clinical team was observed for rare birth defects, such as craniorachischisis and microcephaly. Likewise, among surveillance midwives, the lowest PPV was only observed for craniorachischisis, encephalocele, and cleft lip. The low PPV among midwives, could be attributed to low prevalence of craniorachischisis, encephalocele and cleft lip during the surveillance study period. Additional training and skills-building are needed to help clinical and surveillance staff identify and diagnose rare birth defects. Surveillance teams with capacity similar to the MU-JHU clinical team would also benefit from an additional member with training in clinical genetics, especially for the diagnosis of complicated cases and syndromes.

This study also found that both the MU-JHU clinical team and the surveillance midwives have continuously increased their knowledge of birth defects diagnoses, which is evidenced by the increase in PPVs for most of the birth defects between the period 2015–2018 and 2019–2021. This may be attributed to the trainings that the MU-JHU clinical team and surveillance study staff were required to undergo before participating in surveillance activities, as well as additional training surveillance midwives underwent to conduct bedside birth defect confirmation in the absence of a clinical consultant. For surveillance midwives, strengthening capacity through confirmation by a second midwife could also have helped surveillance midwives gain confidence, experience and expertise in diagnosing birth defects by learning from their colleagues. However, there were some drops in rare defects like craniorachischisis though not statistically significant, potentially attributable to defects being rare, some defects being part of a syndrome and high surveillance midwives turn-over. Also, there may have been over-reporting of cases by surveillance midwives as they are trained to report any abnormality. Diagnoses of some defects may have been complicated by the rarity of a defect, the presence of multiple birth defects, or even a typical presentations of birth defects which would have made accurate identification more of a challenge for surveillance midwives. For some birth defects such as abdominal wall defects and orofacial clefts, the presence or absence of the defect was correctly noted; however, the type of defect may have required additional input by the clinical team at MU-JHU or by birth defects subject matter experts at CDC.

Compared to the period 2015–2018, additional measures were implemented in 2019–2021, to help hospital surveillance staff improve diagnosis and classification of birth defects. For example, during 2019–2021, a greater emphasis was placed on practical sessions and video trainings for anthropometric measurements, which improved skills among hospital surveillance staff for performing physical exams and ruling out abnormal length, weight, and head circumference, especially microcephaly. During this latter period, standard operating protocols were also modified for identification and coding of cases for inclusion in birth defects surveillance. A quality control verification form was put in place, according to identified gaps. Additionally, video conference calls were regularly held between surveillance midwives and MU-JHU clinical team, to provide feedback on incorrectly diagnosed birth defects.

One limitation faced by this surveillance program was the high turnover (37%) among surveillance midwives, as it is not unusual for midwives to change hospitals in resource-limited settings like Uganda [12]. However, the training and skills these midwives gained from working in birth defects surveillance will be taken to other hospitals, which can only strengthen the identification and classification of birth defects in Uganda.

Further improvements in PPV can be attributed to the feedback on birth defects diagnoses and coding, provided by CDC birth defects SMEs to the MU-JHU clinical team. This feedback was periodically given to surveillance study midwives, during meetings at their respective hospitals. The feedback process, for both the MU-JHU clinic team and the surveillance midwives, helped staff gain experience in diagnosing birth defects.

## Conclusion

This study highlighted that birth defects surveillance, by front-line health care workers (midwives) in a low-middle income country like Uganda, is possible when midwives are exposed to comprehensive training, technical support and continuous professional development initiatives. Over the six years of the MU-JHU Research Collaboration Birth Defects Surveillance System, the diagnostic accuracy of the MU-JHU clinical team increased for five defects and decreased for nine defects, but none of the changes in PPV were statistically significant. Among the surveillance midwives, however, diagnostic accuracy increased across several birth defects, four of which were statistically significant. Empowering surveillance midwives to identify and confirm external birth defects is a strategy that is feasible, in a resource limited setting, where there is scarcity of consultants/doctors to provide confirmation.

### Electronic supplementary material

Below is the link to the electronic supplementary material.

Supplementary Material 1

## Data Availability

The dataset used and/or analyzed during the current study are available from the corresponding author on reasonable request.
