# Supplementary material for: Strengthening capacity of health workers to diagnose birth defects in Ugandan hospitals from 2015 to 2021
Source: BMC Med Educ. 2023 Oct 13;23:766. doi: 10.1186/s12909-023-04760-w (PMC10576368; doi:10.1186/s12909-023-04760-w)
Supplement: Supplementary file 1 — Supplementary Material 1 [file 12909_2023_4760_MOESM1_ESM.docx]

**Table 1. Definitions of the different major birth defects**

| **Defect** | **Definition** |
| --- | --- |
| Anencephaly | Total or partial absence of the cranium vault. |
| Craniorachischisis | The presence of anencephaly with a contiguous spine defect without meninges covering the neural tissue (rachischisis). |
| Iniencephaly | An Neural Tube Defect (NTD) involving the occiput and inion, resulting in extreme retroflexion of the head (the cranium is always closed). |
| Encephalocele | Characterized by sac-like protrusions of the brain through a defect in the skull. |
| Spina bifida | An NTD characterized by herniation or exposure of the spinal cord and or meninges thorough an incompletely closed spine. |
| Microcephaly | A congenitally small cranium, defined by occipito-frontal circumference (OFC) >3 standard deviations below the mean for age and sex. |
| Anophthalmia/microphthalmia | Absence of one or both eyes/ one or both eyes are abnormally small. |
| Anotia/microtia | Absent/ small and malformed ear. |
| Cleft palate alone | Fissure in the hard or soft palate in the roof of the mouth. |
| Cleft lip alone | An opening or split in the upper lip. |
| Cleft lip with cleft palate | A gap or split in the upper lip and/or roof of the mouth (palate). |
| Imperforate anus | The absence of a normal anal opening at birth. |
| Hypospadias | Abnormal placement of the urethral meatus on the underside of the penis. |
| Talipes equinovarus | Deformity of the ankle or foot in which the foot is turned inwards and under in a fixed position. |
| Limb reduction | Partial or complete absence of the arm (upper limb) or leg (lower limb). |
| Gastroschisis | An abdominal wall defect characterized by visceral herniation of abdominal organs to the side of the umbilical cord. |
| Omphalocele | An abdominal wall defect characterized by herniation of abdominal contents covered by a thin membrane through the umbilical insertion. |
